# Poroelastic Mechanical Effects of Hemicelluloses on Cellulosic Hydrogels under Compression

**DOI:** 10.1371/journal.pone.0122132

**Published:** 2015-03-20

**Authors:** Patricia Lopez-Sanchez, Julie Cersosimo, Dongjie Wang, Bernadine Flanagan, Jason R. Stokes, Michael J. Gidley

**Affiliations:** 1 Australian Research Centre, Centre of Excellence in Plant Cell Walls, Centre for Nutrition and Food Sciences, Queensland Alliance for Agriculture and Food Innovation, The University of Queensland, Brisbane, Australia; 2 Australian Research Centre, Centre of Excellence in Plant Cell Walls, School of Chemical Engineering, The University of Queensland, Brisbane, Australia; Rensselaer Polytechnic Institute, UNITED STATES

## Abstract

Hemicelluloses exhibit a range of interactions with cellulose, the mechanical consequences of which in plant cell walls are incompletely understood. We report the mechanical properties of cell wall analogues based on cellulose hydrogels to elucidate the contribution of xyloglucan or arabinoxylan as examples of two hemicelluloses displaying different interactions with cellulose. We subjected the hydrogels to mechanical pressures to emulate the compressive stresses experienced by cell walls *in planta*. Our results revealed that the presence of either hemicellulose increased the resistance to compression at fast strain rates. However, at slow strain rates, only xyloglucan increased composite strength. This behaviour could be explained considering the microstructure and the flow of water through the composites confirming their poroelastic nature. In contrast, small deformation oscillatory rheology showed that only xyloglucan decreased the elastic moduli. These results provide evidence for contrasting roles of different hemicelluloses in plant cell wall mechanics and man-made cellulose-based composite materials.

## Introduction

The mechanical properties of plant cell walls are remarkable because they must be flexible and deformable to allow morphogenesis and cell expansion, whilst providing structural integrity and rigidity to the plant. These properties are also of interest in providing inspiration and design rules for the construction of cellulose-based structuring materials for diverse technological uses. The role of specific polysaccharide components in the micromechanical behaviour of plant cell walls is not fully established. The current proposed structural model for the primary plant cell wall describes the wall of dicotyledons and non-commelinid monocotyledons (Type I) as a complex network of cellulose microfibrils and hemicelluloses embedded in a pectin gel network [1–3].

Xyloglucans are typically the main hemicellulose in Type I primary cell walls (10–30% dry weight), and are composed of a cellulose-like backbone of β-(1–4)-linked-D-glucose branched by α-D-xylose molecules, which are further substituted by β-D-galactose and other sugars [4]. The major set of non-cellulosic polysaccharides in the (Type II) primary cell walls of cereals, grasses and related commelinid species are the heteroxylans, consisting of linear chains of β-1,4-linked D-xylose, which in the case of arabinoxylan, are substituted by arabinose on the C-2 and/or C-3 position [5] and can also carry other substituents such as glucuronic acid.

The structural roles of hemicelluloses in the cell wall are not fully established but are considered to include the tethering (crosslinking) of cellulose fibres so as to restrict the ability of fibres to separate laterally. Based on chemical extractability and enzyme accessibility, xyloglucan for example is considered to have three domains in the cell wall; one domain crosslinks or is otherwise between cellulose microfibrils, while another domain binds to the surface of cellulose fibres, and a third domain is entrapped between cellulose microfibrils inside cellulose fibres [6, 7]. The variety of possible interactions between xyloglucan and cellulose also considers a ‘gap-filling’ function for xyloglucan between celluloses fibres [8]. Furthermore it has been inferred from biosynthesis studies that xylans might interact differentially with cellulose depending on the pattern of their substitution [9]. Xyloglucan crosslinks have been proposed to control cell wall mechanics in conjunction with the action of proteins like expansins, and xyloglucan endotransglucosylase, or β-1,4- endoglucanases [10–12]. Recent evidence suggests that a small fraction of the xyloglucan which is involved in the close tethering of cellulose fibres may be particularly important in controlling cell wall strength and extensibility [13, 14].

Plants have the ability to adapt to modifications in their cell wall composition, which has made it very challenging to draw conclusions on the structural role of individual polysaccharides [12]. The use of cell wall analogues based on cellulose hydrogels produced by *Gluconacetobacter xylinus* allows us to systematically study the contribution of defined polysaccharides to the mechanical properties of cellulose composites [11, 15–18]. The microstructure of cellulose/xyloglucan composites [11, 19] is similar to the cross-linked cellulose network in the plant cell wall, and thus the molecular and mechanical properties of such analogues may provide insights into the micromechanics of the plant cell wall and help to identify opportunities for the design of material properties in fabricated cellulose composite materials. Prior studies on the uniaxial and biaxial tensile properties of cellulose/xyloglucan composites concluded that the presence of xyloglucan crosslinks weaken the composites [18] but increased their extensibility [15].

Previous studies investigating the structural basis for the materials properties of plant cell walls and analogues have not, however, taken into account the mechanical coupling between the continuous water phase and the polymer network(s) of the wall. These coupling effects are linked to the incompressibility of water and are dependent on the rate of fluid flow. This biphasic nature of the system is captured by the concept of poroelasticity, which has been shown to account for observed timescales and mechanisms of plant and fungal tissue movements [20], the mechanical and transport properties of cellular cytoplasm [21] and the materials properties of cellulose hydrogels [22]. Here we investigate the mechanics of cellulose/hemicelluloses composites as cell wall analogues using a recently-introduced technique [22] of applying steps in compressive load and relaxation, which includes characterisation of the viscoelasticity after each compressive step using small-amplitude-oscillatory shear. This allows, for the first time, an investigation into the micromechanics of the composite that considers the impact from the flow of water and the subsequent increase in density during compression; we interpret measurements using the concept of poroelastic mechanical behaviour. In comparison to previous reports on cellulose-hemicellulose composites, this study is also the first to apply compressive stresses that approach those present in plant tissues. From this new approach, we discover that composites of cellulose with xyloglucan and arabinoxylan, two model hemicelluloses involved in direct binding and non-specific associations with cellulose respectively, possess qualitatively different behaviours under a wide range of deformation conditions. We discuss how these findings provide new insights into the potential role of hemicelluloses on the micromechanics of the plant cell wall, with implications for the design of cellulose-based composites having tailored material properties.

## Materials and Methods

### Composite preparation

Cellulosic composites were produced following the method described by Chanliaud et al. [23] and Mikkelsen et al. [24]. Briefly the *Gluconacetobacter xylinus* frozen strain ATCC 53524 (Manassas, VA, USA) was cultivated in Hestrin and Schramm medium at pH 5. To prepare 1% (w/v) xyloglucan solution, tamarind xyloglucan (Lot 100402, Megazyme International Ireland Ltd., County Wicklow, Ireland) was dissolved in deionised water at room temperature under sterilised conditions. To produce cellulose/xyloglucan composites, a 1% xyloglucan solution was mixed with double concentrated Hestrin and Schramm medium (1:1) before inoculation, leading to a final xyloglucan concentration of 0.5% w/v. Similar preparation method and concentrations were used for the cellulose/arabinoxylan composites (medium viscosity wheat arabinoxylan, Lot 40302b, Megazyme International Ireland Ltd., County Wicklow, Ireland). Composites were cultivated statically at 30°C for 72 hours in 40 mm diameter containers. After cultivation they were harvested and washed 6 times with ice-cold water under agitation at 100 rpm. Samples were stored in 0.02% NaN_3_ solution and kept at 4°C until further analysis.

### Enzyme treatments

*Endo*-1,4-β-glucanase solution was prepared by mixing 300 μl of *endo*-1,4- β-glucanase (Trichoderma, Megazyme International Ireland Ltd., County Wicklow, Ireland) in 60 mL of 50mM sodium acetate buffer (pH 4.5). *Endo*-1,4- β-xylanase solution was prepared by mixing 500 μl (A. niger, Megazyme International Ireland Ltd., County Wicklow, Ireland) in 60 mL of sodium acetate buffer (pH 4.5). These enzyme concentrations were selected based on the activity of each enzyme and the amount of hemicelluloses present in the composites [11]. Samples were individually placed in a container with 10mL of the enzyme solution and kept in contact overnight at 4°C and 40°C for cellulose/xyloglucan and cellulose/arabinoxylan composites respectively. Then they were washed three times in deionised water for 30 min and kept in 0.02% NaN_3_ solution until further analysis.

### Dry weight measurement

Whole samples were dried in an oven at 105°C for 24 h. The dry matter content was calculated in triplicate by weighing the composites on an analytical balance before and after drying.

### Monosaccharide analysis

Composite compositions were calculated from individual sugar contents on the basis of dry weights. The method of Pettolino et al. [25] was used with some variation. Freeze dried samples (1–5 mg) were hydrolysed with 200 μl 12 M H_2_SO_4_ at 35°C for 1 hour, diluted to 2 M using 3.5 ml water and incubated for a further 3 hours at 120°C. Each sample was cooled, then neutralised using approximately 550 μl of NH_4_OH and centrifuged at 2000 rpm for 10 minutes. An aliquot of 100 μl was collected, 5 μg of internal standard (myo inositol) added and then dried with a stream of nitrogen. The sample was reduced using 200 μl of 20 mg/ml sodium borodeuteride in DMSO at 40°C for 90 min. The reductant was destroyed using 20 μl of acetic acid then acetylated by adding 25 μl 1-methylimidazole followed by 250 μl of acetic anhydride. The sample was allowed to stand for 10 minutes, 2 ml of water was added followed by 1ml dichloromethane (DCM) to extract the alditol acetates, the sample was mixed, centrifuged to aid separation and the DCM phase was then washed twice with 2 ml of water. The DCM was then dried under a stream of nitrogen and reconstituted into 100 μl of DCM, 1 μl of which was analysed on the GC-MS using a high polarity BPX70 column.

### Scanning electron microscopy (SEM)

Uncompressed and compressed samples were freeze-substituted (immediately after compression) according to the method of McKenna et al. [26] with minor modifications. Samples were quickly frozen in liquid nitrogen for 10 s, immediately transferred to a container with 3% glutaraldehyde in methanol at −20°C and kept for 24 h. After that the sample was transferred to 100% methanol at −20°C for a further 24 h. Samples were placed onto a sample holder (120–200 μm, ProSci Tech, Thuringowa, Queensland, Australia) and immediately introduced into absolute ethanol solution at room temperature. Samples were finally dried using a critical point dryer (Tousimis Autosamdri-815, Maryland USA). Dried samples were coated with platinum three times, from the top and from each side, at 10 mA for 100 s (Baltec Med 020 Platinum Coater, Leica microsystems, Wetzlar, Germany). Images were taken using a JSM 6300 electron microscope (JEOL, Tokio, Japan) under the following conditions: acceleration voltage 5 kv, spot size 10 and a working distance (WD) of around 10 mm. All images were taken from the top side of the sample, which is the one in contact with air during production. Images were taken from at least three different positions for each sample and 6 images were taken from each position, with increasing magnifications.

### Mechanical compression test

Measurements were made on a rotational rheometer (HAAKE Mars III Rheometer, Thermo Fisher Scientific, Karlsruhe, Germany) at a constant temperature of 25°C controlled by a Peltier element. Parallel plates with a diameter of 60 mm were used. The upper and bottom plates were coated with fine emery paper (P240/S85, 58 μm roughness).

The samples were handled with tweezers. The composites were placed in the centre of the parallel plates with the help of a stencil. For each experiment the initial gap (distance between top and bottom plates) was adjusted according to the sample thickness.

The behaviour under compression was studied at three different strain rates 1, 10 and 100 μm/s to investigate the response of the composites to short and long time scales of deformation [27]. Samples were compressed from their initial thickness to the narrowest possible gap (typically 300–500 μm) achievable within the constraints of the normal force transducer (50 N) of the instrument. Between 6 and 9 replicates were measured.

### Small amplitude oscillatory shear deformation test (SAOS)

Small deformation experiments were carried out in the same instrument and with the same plates as the compression tests. The samples had a diameter smaller than that of the plates therefore both the shear and stress factors were adjusted [28]. In order to identify the linear regime of the composites, amplitude sweep tests were performed at frequencies of 0.1 rad/s and 10 rad/s over a stress range of 1 to 100 Pa. From this, a shear stress of 1 Pa is selected for measuring the linear viscoelastic properties of all three samples. Experiments were carried out in duplicate.

### Axial compression and SAOS (cycle test)

To follow the change in viscoelastic properties as a function of confinement and polymer concentration, we carried out a test where an axial compression was applied followed by a small amplitude oscillatory shear test in 100 μm steps [22]. During the axial compression, samples were compressed 100 μm at a constant speed of 1, 10, or 100 μm/s. At each gap an oscillatory shear test was performed at a frequency of 0.016 rad/s and at a stress of 1Pa. These two tests were reproduced several times from the initial thickness of the samples to the narrowest possible gap. Experiments were performed at least in triplicate. The samples were weighed and their diameter measured before and after the test.

### Solid State NMR

To examine changes in cellulose crystallinity ^13^C CP/MAS NMR experiments were performed at a ^13^C frequency of 75.46 MHz on a Bruker MSL-300 spectrometer. Uncompressed samples were blotted dry whilst compressed samples were used without modification before being packed in a 4 mm diameter, cylindrical, PSZ rotor with a KelF end cap. The rotor was spun at 5 kHz at the magic angle (54.7°). The 90° pulse width was 5 μs and a contact time of 1 ms was used for all samples with a recycle delay of 3 s. The spectral width was 38 kHz, acquisition time 50 ms, time domain points 2 k, transform size 4 k and line broadening 50 Hz. At least 2400 scans were accumulated for each spectrum. Spectra were referenced to external adamantane. Using single pulse (direct polarization) excitation with magic angle spinning (SP/MAS) the mobile components of the hydrogels were observed. The recycle time was 60 s and 20 k spectra were accumulated.

## Results

### Characterisation of cellulose/hemicelluloses composite hydrogels

The characteristics of cellulose only (C), cellulose/xyloglucan (CXG) and cellulose/arabinoxylan (CAX) composite hydrogels are listed in Table 1. Hydrogels were harvested after 72 h fermentation, and were disks with diameters of ca. 40 mm corresponding to the diameter of the containers used for cellulose synthesis. C had the greatest thickness, the lowest dry weight, and the lowest polysaccharide content. The dry weight was similar for C and CXG but higher for CAX. The polysaccharide concentration in the hydrogels for CXG and CAX was double that of C. Furthermore the initial density of CXG was higher than C samples. These differences demonstrate the active effects that inclusion of XG or AX in the fermentation media have on the biosynthesis of cellulose hydrogels by *G*. *xylinum*.

**Table 1:** Dimensions, dry weight, polysaccharide concentration, and density of C, CXG and CAX composites.

Sample	^1^Thickness (mm)	^2^Dry weight (mg)	^2^Polysaccharide concentration (% w/w)	^2^Density (g/cm^3^)
C	2.8 ± 0.2	25 ± 0.3	0.7 ± 0.05	0.007 ± 0.001
CXG	1.3 ± 0.2	29 ± 0.1	1.4 ± 0.3	0.017 ± 0.001
CAX	1.9 ± 0.1	37 ± 0.2	1.4 ± 0.2	0.015 ± 0.001

^1^average and standard deviation of 12 samples.

^2^average and standard deviation of 3 samples.

Monosaccharide analysis of washed composites showed 37.5% incorporation of XG corresponding to a ratio cellulose: xyloglucan of 1:0.6. This composition is similar to the ones obtained previously by Whitney et al. [29]. Composites containing arabinoxylan had a composition of 53.1% arabinoxylan corresponding to a ratio cellulose: arabinoxylan 1:1.13, more than 4 times higher than the one reported earlier [30] following less extensive dissolution of AX prior to *G*.*xylinus* fermentation.

**Fig 1:**
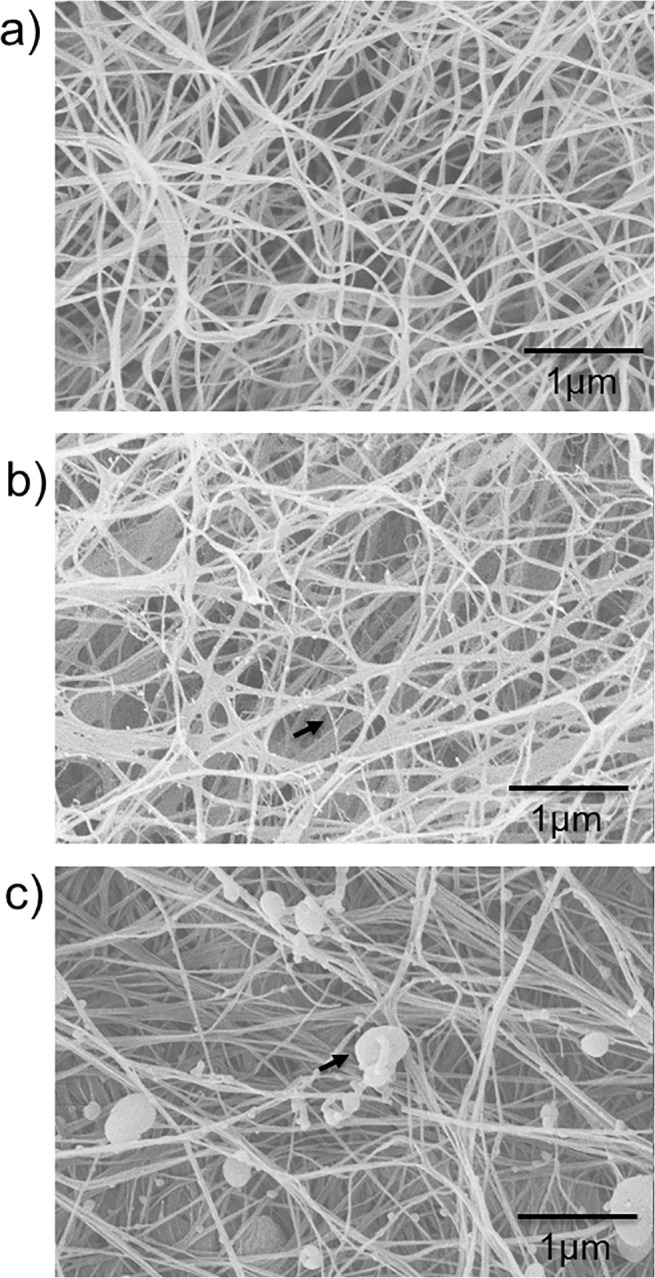
Scanning electron micrographs of a) cellulose only, b) cellulose/xyloglucan and c) cellulose/arabinoxylan. Xyloglucan crosslinks and arabinoxylan aggregates can be observed in the composites as indicated by black arrows.

Fig. 1 shows the structure of the hydrogels prior to compression. C composites appeared to be a network of highly interconnected and randomly oriented long cellulose fibres (Fig. 1A). The inclusion of xyloglucan in the fermentation medium during bacterial cellulose production showed the presence of thin strands between thicker fibres, which we consider to be tethers of XG between the cellulose fibres (Fig. 1B), in agreement with previous studies [16]. In the CAX composites no strands are observed, but instead aggregates of less than 1 μm in size were locally deposited onto the cellulose fibres (Fig. 1C); similar microstructures have been previously reported for CAX composites [30]. In addition, they were still present after extensive washing of the samples, indicating that arabinoxylan interacts with the cellulose fibres. CXG hydrogels were approximately half the thickness of cellulose-only samples produced after the same fermentation time, likely due to the effect of xyloglucan crosslinks bringing cellulose fibres closer together leading to a more compact structure. This is also reflected in the higher density of these composites compared to cellulose only and cellulose/arabinoxylan hydrogels (Table 1).

In Table 2 it can be observed that the presence of xyloglucan decreased the apparent NMR-estimated crystalline content from 87% to 64%. These estimated values are similar to those previously found by Whitney et al. [16]. This decrease is proposed to be the result of the direct association between xyloglucan and cellulose microfibrils prior to final assembly of the cellulose fibre. However, in the presence of xyloglucan, it is likely that the level of non-crystalline cellulose is overestimated due to the overlap of C-4 xyloglucan and non-crystalline cellulose between 78 and 84ppm. It was also observed that the ratio of I_α_ to I_β_ cellulose crystalline allomorph changes when xyloglucan is incorporated with the percentage of I_β_ allomorph increasing (Table 2), a measurement which is based on crystalline cellulose C-4 signals at 88–91 ppm which do not overlap with XG peaks. In contrast, arabinoxylan had no detectable effect on either the crystalline content or the I_α_ to I_β_ allomorph ratio, consistent with the lack of specific interactions of AX with cellulose. Treatment of CAX with *endo-*xylanase also had no effect on cellulose crystallinity or I_α_ to I_β_ ratio of CAX; the apparent increased crystallinity of CXG after treatment with *endo*-glucanase is probably a result of the decrease in XG overlapping the non-crystalline cellulose, with the I_α_ to I_β_ ratio unaltered (Table 2).

**Table 2:** Percentage of total crystallinity and component crystalline allomorphs in cellulose hydrogel composites.

Sample	% crystalline (± 2)	% I_α_ (± 2)	% I_β_ (±2)
C	80	62	38
CXG	64	39	61
CAX	78	60	40
*Endo*-glucanase CXG	73	38	62
*Endo*-xylanase CAX	79	60	40

**Fig 2:**
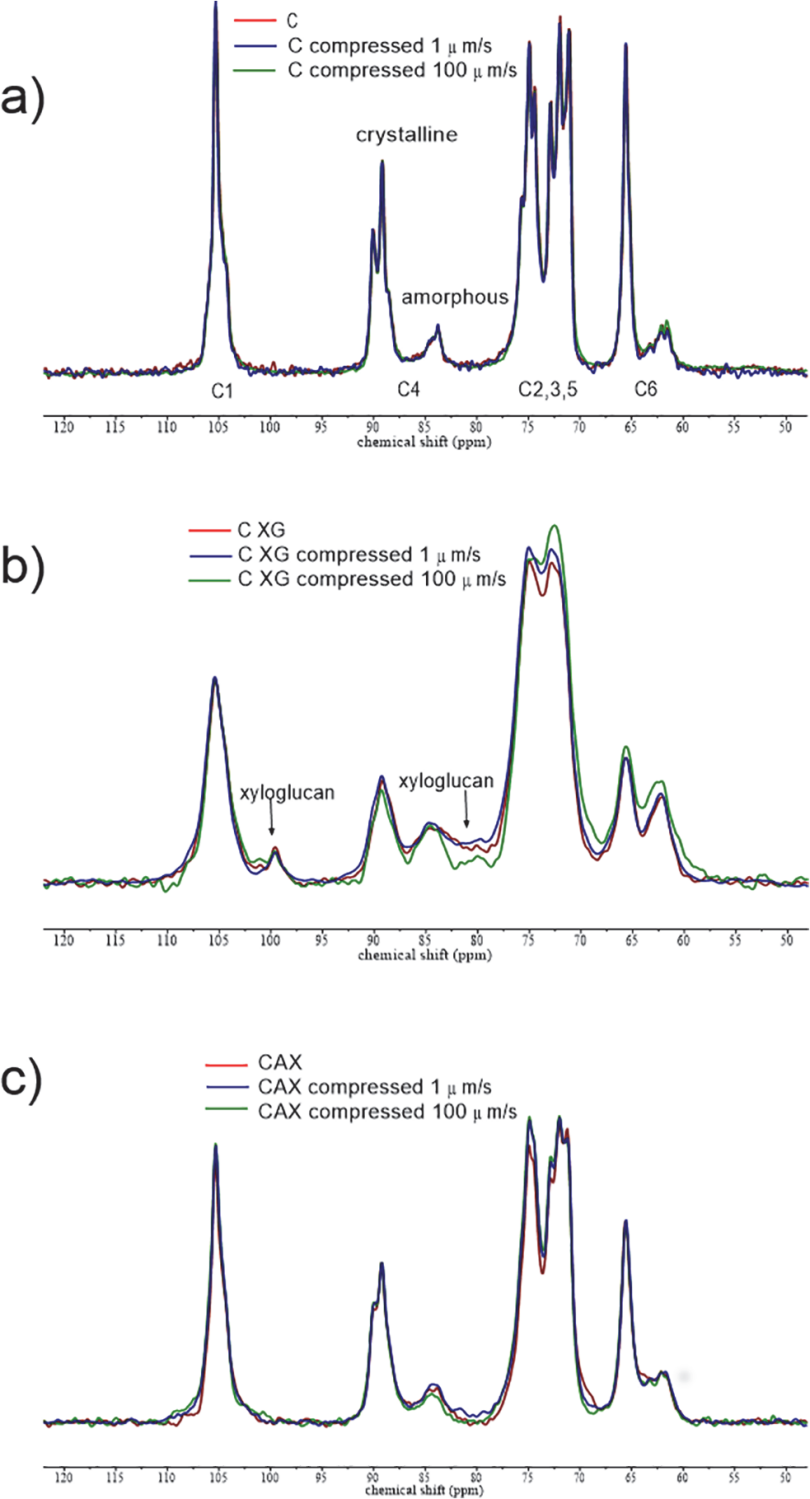
^13^C CP/MAS spectra of a) C, b) CXG and c) CAX hydrogel composites. Uncompressed and compressed samples at 1μm/s and 100 μm/s. The peak for xylose C-1 in rigid xyloglucan can be seen at 99.5 ppm.

The presence of rigid segments of xyloglucan in CXG composites is indicated in Fig. 2 from the ^13^C CP/MAS NMR spectrum, which shows a peak at 99.5 ppm (Fig. 2B) due to the C-1 of xylose [31]. From integration of the xylose C-1 signal to the signal centred at 105 ppm due to cellulose C-1 and XG glucose and galactose C-1, the fraction of XG detected in the CP/MAS spectrum can be calculated to be 18% of the total composite or 48% of the XG (total XG is 37.5% of the composite). Xyloglucan but not cellulose is also observed in the ^13^C SP/MAS NMR spectrum that is attributable to mobile segments of xyloglucan. Thus the structure of CXG can be described as containing 18% rigid XG, 19.5% mobile XG and 62.5% (rigid) cellulose. The ^13^C CP/MAS spectrum of the CAX composite was indistinguishable from that of C (Fig. 2C). However, the ^13^C SP/MAS spectrum revealed 2 peaks in the C-1 region typical of arabinoxylan: xylose at 102.4 ppm and arabinose at 108.5 ppm. Therefore, although the AX is incorporated irreversibly within the composite, it retains internal mobility despite being associated with the surface of the cellulose scaffold.

### Mechanical properties of hydrogel composites during compression at different compressive strain rates

The normal force (F_n_) and gap is measured during the compression of the cellulose hydrogels. The normal stress and compression strain is given by:

$$\mathrm{Normal stress}=F_{n}/A$$ (1)

$$\mathrm{Compression strain}=\left(h_{i}-h_{f}\right)/h_{i}$$ (2)

The surface area of the samples is A = 1300 mm^2^, while h_i_ and h_f_ represent the initial composite thickness (height) and the thickness (height) at each gap respectively.

**Fig 3:**
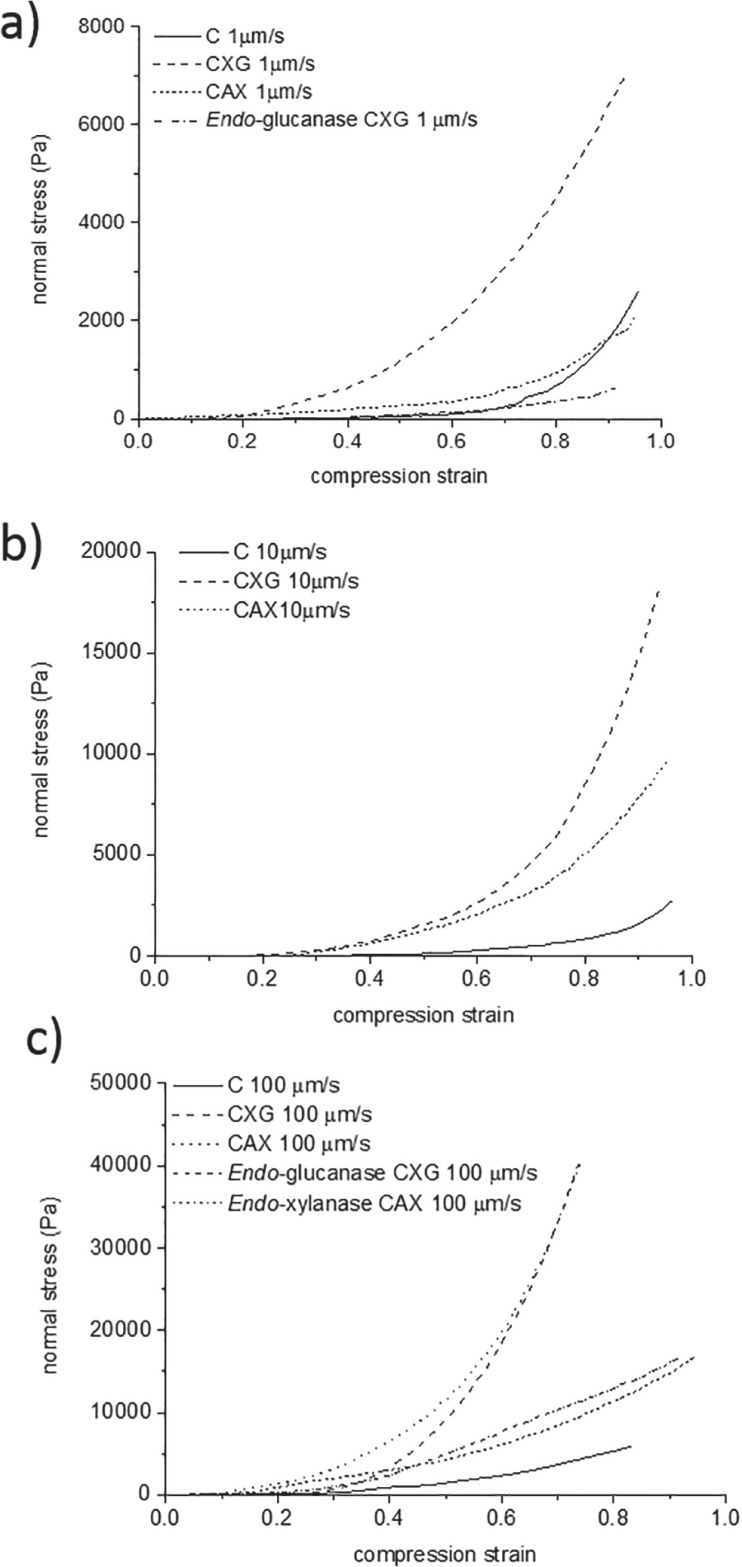
Representative compression curves of cellulose only (C), cellulose/xyloglucan (CXG) and cellulose/arabinoxylan (CAX) composites. a) Slow speed 1 μm/s, b) intermediate speed 10 μm/ and c) fast speed 100 μm/s. Results of enzymatically treated composites are also included for the slowest and fastest speeds.

Samples were compressed at three different speeds; 1, 10 and 100 μm/s from their initial thickness to 500μm. Fig. 3 shows representative normal stress—compressive strain curves. All three cellulose hydrogels showed a great degree of reproducibility within and between batches; small variability is expected due to the biological nature of the samples. During compression of the samples, water is squeezed radially out of the porous cellulose network. We recently showed that C has a Poisson ratio of approximately zero, indicating that it compresses without significant radial expansion [22]. Normal stresses arise during the compression due to both the resistance to water flow, which is dependent on the porosity, and the structural mechanics, both of which alter continuously during compression. CXG hydrogels showed the largest resistance to compression at all three compression speeds tested. CAX hydrogels were very similar to C at the slowest strain rate; however at 10 μm/s, they are similar to the CXG hydrogels and no differences were observed between CXG and CAX hydrogels at the fastest strain rate of 100 μm/s, with both being clearly distinct from C (Fig. 3).

The axial modulus (stiffness) of the fully compressed sample is estimated from the linear slope of the normal stress—strain curve at large strains. At slow strain rates, the presence of the hemicelluloses does not have a significant effect (ANOVA single factor, P>0.05) on the apparent stiffness of the composites with values of ca. 4.5, 10 and 12.5 kPa for CAX, CXG and C respectively. However at the fastest strain rate of 100 μm/s, the composites containing xyloglucan or arabinoxylan were approximately 10 times stiffer than the cellulose only samples going from 10 kPa to approximately 100 kPa.

The initial densities of the composites in Fig. 3 were slightly different (Table 1). To compare the compression behaviour of composites with the same initial density, we calculated the volume of samples compressed to different gaps during the cycle test. The diameter of the samples after total compression were 41.2 ± 0.3 mm, 40.9 ± 0.15 mm and 41.6± 0.16 mm for cellulose only, cellulose/xyloglucan and cellulose/arabinoxylan composites respectively. These diameters are not significantly different compared to the initial diameter before compression (Table 1). Thus the volume could be calculated for each gap, or sample thickness, using volume = π *r^2^ * h_f_, where h_f_ is the thickness at each gap and *r* is the sample radius.

**Fig 4:**
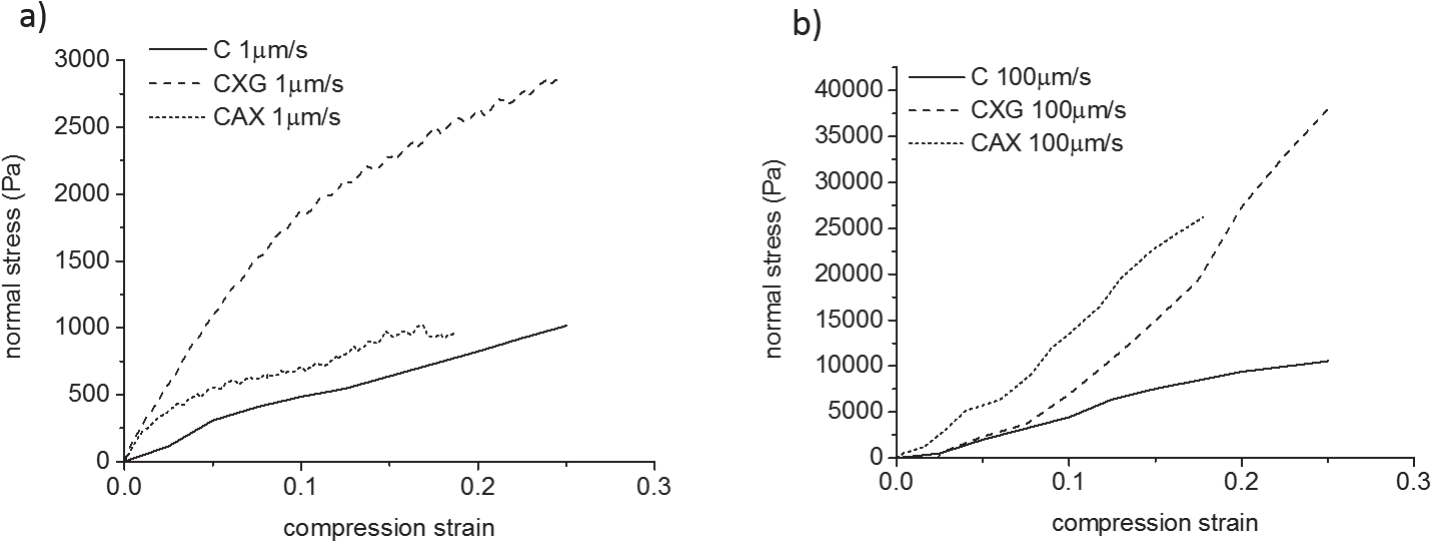
Representative normal stress-compression strain curves. Composites with the same density of 0.05 g/cm^3^ at a) the slowest and b) the fastest strain rates for a compression step of 100 μm.

We selected samples with polysaccharide density of 0.05 g/cm^3^, corresponding to initial thicknesses of 400 μm for C and CXG and 500 μm for CAX and concentrations of ca. 3.5%–4.5% w/w. Furthermore we compared small compression steps of 100μm to investigate the behaviour at small strains (Fig. 4).

As can be observed in Fig. 4, composites with the same density showed similar trends to those observed for samples with different initial density (Fig. 3). This indicates that the mechanical response of the hydrogels is not only controlled by the composite density. At 1μm/s, composites containing xyloglucan were stronger than cellulose only samples whereas the presence of arabinoxylan did not have a significant effect on the mechanical response (Fig. 4A). As the compression speed was increased, the presence of hemicelluloses (arabinoxylan or xyloglucan) markedly increased the resistance to compression of the composites (Fig. 4B).

Furthermore the micromechanical behaviour under these confined conditions showed qualitative differences as a function of the strain rate. At slow strain rates (long time scales) all three samples behaved as viscoelastic materials (Fig. 4A). First a linear elastic region was observed up to strains of ca. 0.05 (5%) for C and CAX and higher strains of ca. 0.1 (10%) for CXG, followed by a non-linear plastic deformation. In contrast, at fast compression strain rates (short time scales), composites containing xyloglucan or arabinoxylan showed only a limited elastic region (Fig. 4B). In a similar fashion the elastic behaviour of cellulose only samples was extended to higher strains of ca. 0.1 (10%) at the fastest strain rate compared to the slowest strain rate.

### Enzyme effects on mechanical properties

Xyloglucan and arabinoxylan were depleted from CXG and CAX composites using *endo*-glucanase and *endo*-xylanase respectively. Monosaccharide analysis showed a content of 16.7% xyloglucan after the enzymatic treatment (corresponding to 55% depletion of xyloglucan from the composite) and near complete removal of arabinoxylan, with only 1% remaining of the 53% prior to enzyme treatment. The ^13^C CP/MAS spectra of CXG following *endo*-glucanase treatment (S1 Fig.) revealed that the amount of XG immobilised by the cellulose scaffold had decreased by approximately 60% to 6.7% rigid XG and the mobile component decreased by almost 50% to 10%. The ^13^C SP/MAS spectrum (S2 Fig.) was identical to that of the untreated sample confirming the presence of xyloglucan but not cellulose. It is interesting that enzyme treatment reduces the levels of rigid and mobile segments of XG to approximately equal extents.

*Endo*-xylanase treatment of CAX did not affect the structure of the cellulose or result in any immobilisation of AX as judged by ^13^C CP/MAS NMR (S3 Fig.). The characteristic arabinoxylan peaks at 102.4 ppm (C-1 xylose) and 108.5 ppm (C-1 arabinose) were also completely removed from the ^13^C SP/MAS spectrum (S4 Fig.).

The mechanical behaviour of CXG hydrogels after *endo*-glucanase treatment was closer to C hydrogels, composites became weaker showing less resistance to the compression than before the enzymatic treatment. This effect was observed for both the slowest and the fastest strain rates (Fig. 3A, 3C). Similarly for cellulose/arabinoxylan composites the increase in strength observed at fast strain rates was reduced after substantial removal of arabinoxylan by xylanase (Fig. 3C).

### Viscoelasticity of cellulose-hemicellulose hydrogels

**Fig 5:**
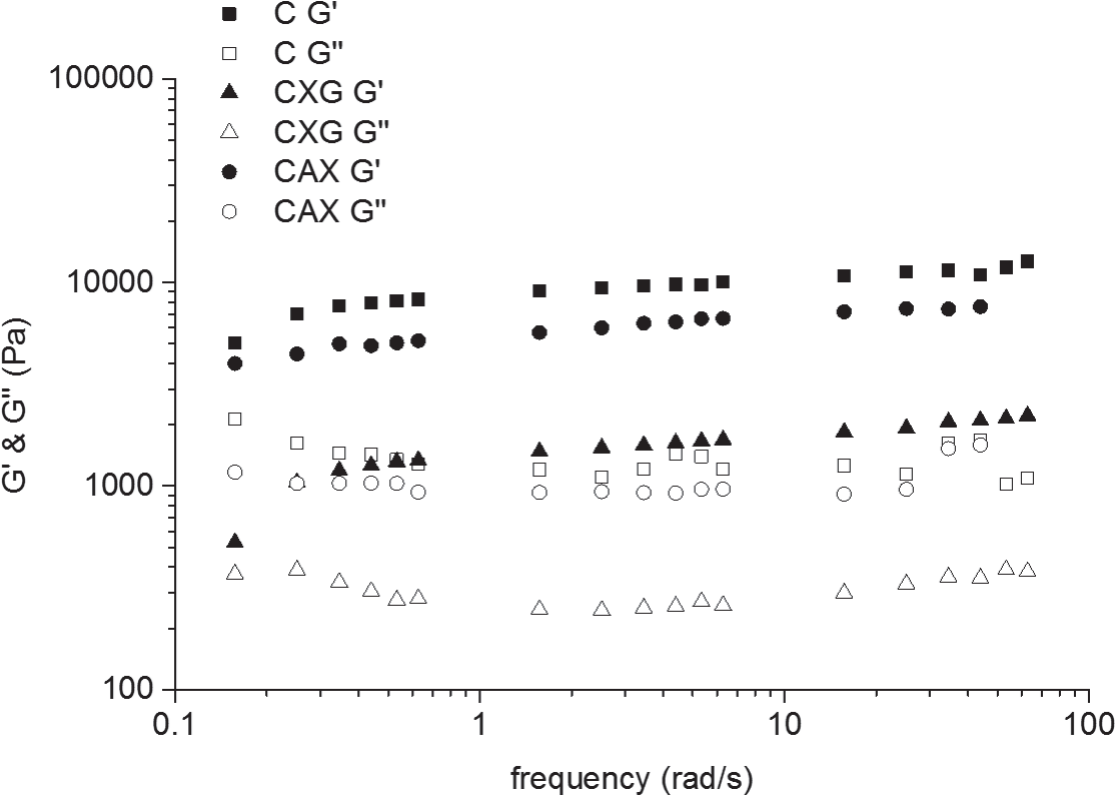
Viscoelastic properties of cellulose hydrogels compressed to 3.5–4.5% polysaccharide concentrations and density 0.05g/cm^3^ as a function of frequency. Cellulose (squares), cellulose/xyloglucan (triangles) and cellulose/arabinoxylan (circles). Full symbols represent the elastic modulus G’ and empty symbols represent the viscous modulus G”.

Small amplitude oscillatory shear (SAOS) experiments are performed to obtain an insight into the composites’ viscoelastic properties under unperturbed conditions. The moduli were relatively independent of frequency for all three samples, and the storage (elastic) modulus G’ was larger than the loss (viscous) modulus G” (Fig. 5), in agreement with previously reported data [18].

The cycle test, a sequence of axial compressions and SAOS [22], allowed us to measure the viscoelastic properties of the composites as a function of the sample thickness. The polysaccharide concentrations at each sample thickness were calculated as described elsewhere [22], briefly we assumed that the change in volume of the composites at each gap is due to water loss and therefore, since the initial water content of the samples is known, the water content at each gap can be calculated. The concentration of polysaccharides is then:

%polysaccharide concentration=dry weight/(dry weight+water content) (3)

**Fig 6:**
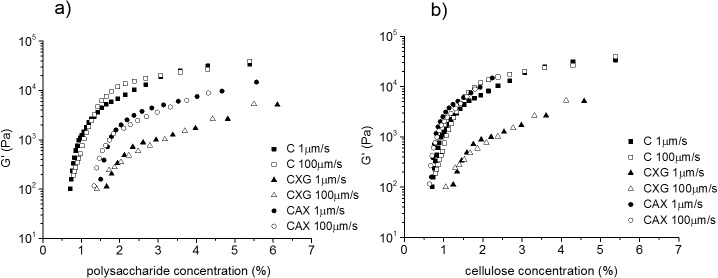
Elastic modulus G’ of cellulose only (C), cellulose/xyloglucan (CXG) and cellulose/arabinoxylan (CAX) hydrogels. As a function of a) total polysaccharide concentration and b) cellulose concentration at different strain rates.

During compression, we were able to concentrate the hydrogels up to 10 times to reach polysaccharide concentrations of around 6%. As can be observed in Fig. 6A, G’ increased with polysaccharide concentration, indicating a more solid-like hydrogel. Interestingly there were no significant differences in the final G’ values at different compression speeds. In contrast with the effect observed during the compression experiments, the presence of xyloglucan weakened the hydrogels under SAOS, reflected in G’ values of 200 to 40000 Pa for C samples and 10 to 5000 Pa for CXG hydrogels. Similar effects were observed when arabinoxylan was present, however the effect of arabinoxylan was not as marked as for xyloglucan with G’ values between 100 and 15000 Pa. When these same G’ values were plotted as a function of cellulose concentration (Fig. 6B) it is observed that CXG composites were weaker than C samples, however for the same cellulose concentrations the presence of arabinoxylan showed no effect on the small deformation behaviour of the hydrogels. Analysis of the data in Fig. 6, showed that the hydrogels have a modulus/concentration relationship close to that expected for biopolymer networks with G'∝c^2^ [32].

### Microstructural changes during compression

**Fig 7:**
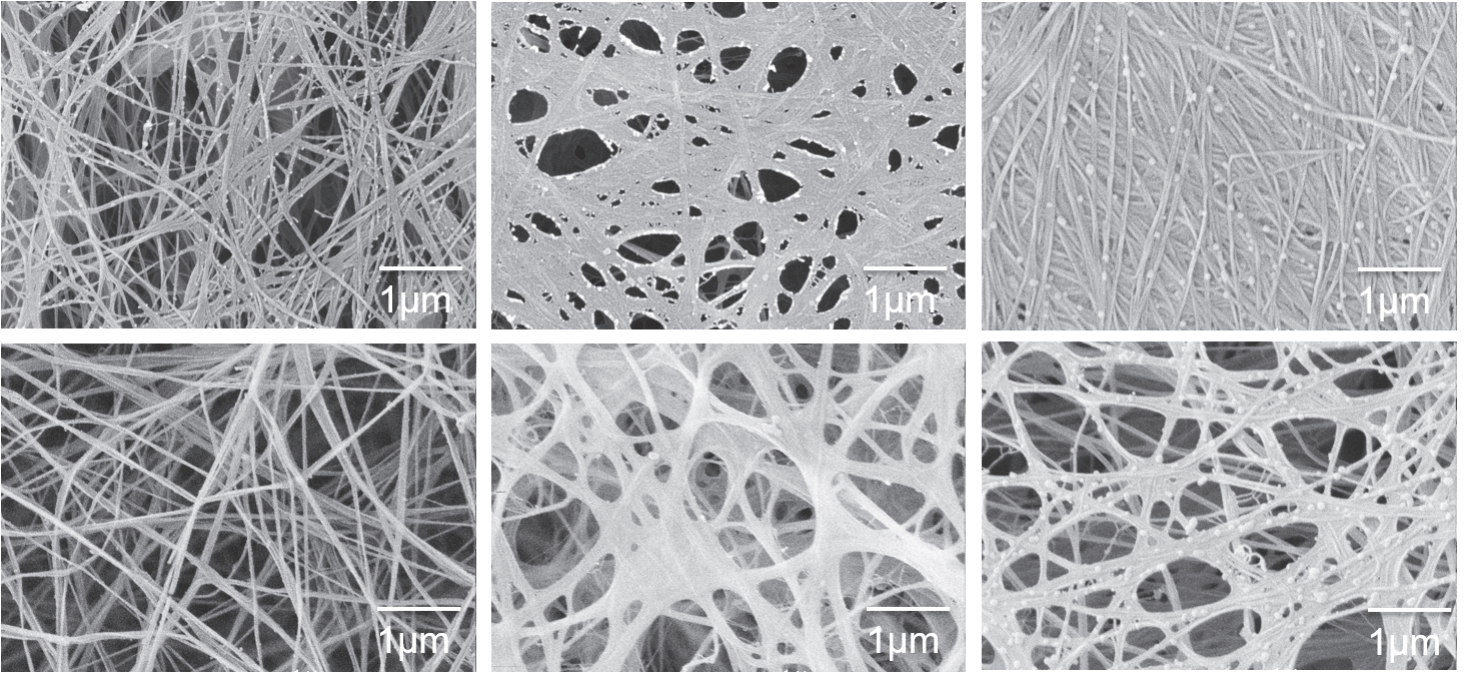
Scanning electron micrographs of hydrogels after total compression (~6% total solids) at 1 μm/s (top row) and 100 μm/s (bottom row). From left to right, cellulose only C, cellulose/xyloglucan CXG and cellulose/arabinoxylan CAX composites.

Visualisation of the architecture after compression (Fig. 7) revealed a higher degree of collapse or densification of the structure at slow strain rates compared to fast rates. This effect was more pronounced when the hemicelluloses were present, especially in the case of arabinoxylan composites. In all the hydrogels, cellulose fibres were aggregated after compression, reflected in thicker bundles, with the exception of the fast compressed cellulose only, which showed hardly any aggregation [22]. In the case of cellulose/xyloglucan hydrogels the thin-strands of xyloglucan tethering adjacent cellulose fibres could no longer be identified after compression. Arabinoxylan aggregates were still deposited on the surface of the cellulose fibres in the cellulose/arabinoxylan composites after compression at both speeds.

The cellulose only ^13^C CP/MAS spectra were unchanged following compression as seen in Fig. 2A. Although there were minor differences in the ^13^C CP/MAS spectra of xyloglucan and arabinoxylan hydrogels following compression, the differences in calculated crystallinity were within the experimental error (Table 2). There was no observable change in the level of rigid xyloglucan (Fig. 2B) or any evidence of rigid arabinoxylan (Fig. 2C) in ^13^C CP/MAS spectra after compression. The ^13^C SP/MAS spectra of compressed hydrogels showed that mobile arabinoxylan and xyloglucan were still present (S5 Fig.).

## Discussion

In the last few decades, significant advances have been made in elucidating the molecular composition and microscopic features of the primary plant cell wall, however the role of individual biopolymer components on the physico-chemical and mechanical properties of the cell wall is still poorly understood [33]. This is mainly due to the limited knowledge of plant cell wall architecture and the adaptation capacity of plants which can retain material properties of the wall in mutant genotypes lacking individual components by using different polysaccharides [34, 35]. In this work we approached this issue by using cellulose based hydrogels produced by *Gluconacetobacter xylinus*. This organism is able to produce cellulose with a high degree of purity [36], and polysaccharides can be added to the incubation medium which may spontaneously assemble into the cellulose scaffold forming composites with architectural and molecular similarity to the primary plant cell wall [16, 23, 24]. Such composite materials have been widely studied under uniaxial and biaxial tensile tests [11, 15, 16, 18, 37]. We now extend those studies by applying external mechanical pressures to densify the composites and mimic the compressive stresses experienced by the cell wall as a result of the osmotic gradients due to water movement in /out of the cell (turgor pressure). In this study cellulose- hemicellulose composites were subjected to pressures of up to 0.04 MPa, the largest values corresponding to the fast compression speeds. We propose that the normal stresses experienced by the cellulosic composites approximate those stresses experienced in cell walls from appressed cells due to turgor pressures of 0.5–1 MPa. [38] We were able to obtain composites with high hemicellulose incorporation, around 37% for xyloglucan and 53% for arabinoxylan, comparable to values found in plant cell walls. Compression of the composites led to total polysaccharide concentrations of up to 6%, approaching estimated concentrations in primary walls of 10–30%. Scanning electron microscopy confirmed the presence of xyloglucan as tethers/crosslinks between cellulose fibres and arabinoxylan aggregates interacting with cellulose without forming cross-links, and ^13^C MAS NMR showed that arabinoxylan in composites had segmental flexibility whereas approximately half of the xyloglucan in composites retained segmental flexibility while the other half was effectively immobilised, presumably by direct binding of segments to cellulose fibres.

### Hemicelluloses do not prevent the collapse of cellulose networks

The presence of the hemicellulosic polysaccharides did not prevent the collapse of the cellulose network during compression; on the contrary SEM micrographs (Fig. 7) showed an apparently larger degree of densification in composites containing either xyloglucan or arabinoxylan. All three composites contained similar overall cellulose content, the main load-bearing component. The greater collapse or densification of the composites containing xyloglucan or arabinoxylan suggests that each of these two hemicelluloses which associate with cellulose surfaces in the uncompressed state can cause adhesion of cellulose fibres on compression.

### Xyloglucan and arabinoxylan have different effects on mechanical properties depending on the time scale of deformation

Commonly depicted models of the plant cell wall [1, 2] suggest that the hemicelluloses contribute to the load bearing properties of the hydrated cellulose network. In contrast, Cavalier et al. [39] have recently shown that the absence of xyloglucan in *Arabidopsis* mutants led to a decrease in strength of the plant under tensile test, in agreement with the effect observed in cellulose/xyloglucan composites [15, 18]. However Park and Cosgrove [12] recently showed that a xyloglucan-deficient mutant (xylosyltransferase1/xylosyltransferase2 [xxt1/xxt2]) of *Arabidopsis thaliana* had weaker cell walls under tension, but they were stronger in other forms of deformation that mimic the complex processes of wall creep, stress relaxation, and extension growth. Their results suggest that pectins and xylans take on a larger role in the absence of xyloglucan; furthermore they proposed a cell wall model where crosslinks formed by xyloglucan are not present as a load-bearing fibril extended between cellulose fibres, but that the mechanical effect of XG is limited to a small fraction that effectively binds regions of cellulose microfibrils together [14].

In our studies the presence of xyloglucan increased the mechanical strength or resistance to compression at all strain rates tested (Figs. 3, 4). CXG composites were able to withstand compression strengths at least 3 times larger than C; this effect was even more pronounced at fast strain rates (short time scales) showing an effective modulus up to 8 times larger. Similar behaviour under compression has been reported for other cellulose composites containing polyacrylamide [40]. In contrast to the increase in modulus under compression, CXG composites are much weaker than cellulose alone under uniaxial or biaxial tension [11]. This difference in behaviour of xyloglucan composites under compression and tension is due to the orientation of the cellulose microfibrils and differences in how the water can flow out of the structure under load. Under the compression test studied here, water can only flow out of the sides of the hydrogel disks which have a low surface area and porosity whereas in tensile tests water can flow from all surfaces of the test pieces. Hence the contribution of fluid confinement to the deformation is negligible in tensile tests, while under the compression tests reported here water raises the internal pressure of the system which increases significantly the apparent stiffness of the cellulose hydrogel. As shown by the near-zero Poisson ratio, under the compression test there is no significant radial extension of the hydrogel which indicates that the network exhibits greater resistance to deformation in the longitudinal direction of the fibres.

During compression the fibres may buckle, bend or slip at connexion points due to cellulose entanglements or xyloglucan crosslinks. In CXG, SEM and mechanical tests suggest that xyloglucan crosslinks have less resistance to compression compared to cellulose entanglements, allowing the cellulose fibres to collapse onto each other increasing the density and compression strength of the network. In addition, the presence of the hemicelluloses might affect the interstitial fluid drag, raising the internal pressure caused by water and increasing the stiffness of the composites; this effect would be more apparent at fast compression speeds, as is observed (Figs. 3 and 4).

In clear contrast to xyloglucan, the presence of arabinoxylan did not affect the micromechanical behaviour at slow strain rates /long time scales (Figs. 3 and 4), but did at short time scales. In the case of arabinoxylan, no crosslinks were observed (Fig. 1C) and no changes to the cellulose crystallinity were measured (Table 2). Therefore we can assume that a similar number of cellulose entanglements are present compared to cellulose only samples and thus we observe similar compression strengths. As the strain rate increases, and consequently the time of the deformation shortens, the water present in the composites plays a more significant role in generating a high internal pressure and leading to higher normal stresses. The presence of the non-cellulosic polysaccharides make it more difficult for the water to flow due to their viscoelasticity, independently of how they interact with the cellulose scaffold, which led to similar behaviour for the CXG and CAX composites under rapid compression.

These results suggest different potential roles for hemicellulosic components in governing the micromechanics of the plant cell wall. While xyloglucan (and other cellulose-binding and cross-linking polysaccharides from e.g. the mannan family [17]) has the potential to play a role in making the plant cell wall more resistant to compressive stresses at both long and short times of deformation, arabinoxylan (and other more loosely-associated polysaccharides) may contribute in a similar fashion at short time deformations such as in responding to a sudden impact on the cell wall.

### Micromechanical behaviour is qualitatively different depending on the time scales of deformation

Mechanical testing of compressed and hence concentrated (up to 6% w/v polysaccharide) composites (Fig. 4) with densities of around 0.05 g/cm^3^ showed a qualitatively different behaviour for slow strain rates (long time scale) than for fast strain rates (short time scale). All three composites behaved as viscoelastic materials at long time scales showing an initial (low strain) linear elastic region followed by a plastic deformation. The presence of xyloglucan extended the elastic region to larger strains.

Interestingly at short timescales of deformation the presence of hemicelluloses led to a mainly elastic behaviour. The elastic behaviour was also predominant in the cellulose only samples being extended to values double those for slow strain rates. We propose that at slow strain rates mechanical behaviour is due mainly to microstructural contributions while at fast strain rates the contribution from constrained fluid becomes dominating, leading to mainly elastic behaviour throughout. The effect of hemicelluloses on fluid movement is suggested to be the result of the presence of polysaccharides between cellulose fibrils, irrespective of whether the polysaccharide is tethered to the cellulose (CXG) or loosely associated with the surface of fibrils (CAX), increasing the hardness of the composites under compression. This together with the motions of the polysaccharide chains themselves will contribute to the mechanical response.

These results suggest a key role for water in plant cell wall mechanics which would be especially significant at short times of deformation, assisting the plant to resist sudden deformations.

### Xyloglucan and arabinoxylan show different effects on viscoelastic properties during small deformation

Under small deformation oscillatory test conditions, the general behaviour with e.g. G’> G”, showed that there is little difference in behaviour independent of the hemicellulose present. The presence of xyloglucan weakened the composites at small deformations, reflected in lower G’ values. However arabinoxylan, at similar cellulose contents (Fig. 6), had no effect on the small deformation properties. The small deformation behaviour of cellulose composites has been hypothesised to be controlled by the number of cellulosic entanglements, in a similar fashion to networks of flexible rods [18]. Our results are consistent with this hypothesis, we proposed that there will be a lower number of effective entanglements in composites containing xyloglucan, due to the fact that short crosslinks will force the fibres to be aligned into certain orientations, decreasing the points for possible entanglements. Arabinoxylan on the other hand is not expected to affect the number of entanglements, since it is not found crosslinking the cellulose fibres but loosely associated with their surfaces.

## Conclusions

The studies reported here demonstrate that the mechanical effects in cellulose composites of two types of hemicelluloses representing cross-linking (xyloglucan) and surface adsorption (arabinoxylan) to cellulose are different, and vary with timescale, mode and amplitude of deformation. The role of restricted fluid flow becomes more pronounced at higher concentrations and applied deformation rates. It is likely that this diversity of mechanical properties plays important roles in both protecting plants from sudden mechanical insults and also allowing long time cell expansion from similar composite structures.

## Supporting Information

S1 Fig ^13^C CP/MAS of CXG before (a) and after (b) *endo*-glucanase treatment note the reduction of the xylan peak at 99.5 ppm due to the C-1 of xylose. (TIF)

S2 Fig ^13^C SP/MAS of CXG before (a) and after (b) *endo*-glucanase treatment. Xyloglucan still present following enzyme treatment.
(TIF)

S3 Fig ^13^C CP/MAS of CAX before (a) and after (b) *endo*-xylanase treatment. Cellulose is unchanged following enzyme treatment.
(TIF)

S4 Fig ^13^C SP/MAS of CAX before (a) and after (b) *endo*-xylanase treatment. C-1 xylose peak at 102.4 ppm being completely removed and the arabinose peak at 108.5 also no longer evident.
(TIF)

S5 Fig ^13^C SP/MAS of CAX and CXG before and after compression. (TIF)
