# Supplementary figures and images for: Poroelastic Mechanical Effects of Hemicelluloses on Cellulosic Hydrogels under Compression
Source: PLoS One. 2015 Mar 20;10(3):e0122132. doi: 10.1371/journal.pone.0122132 (PMC4368770; doi:10.1371/journal.pone.0122132)

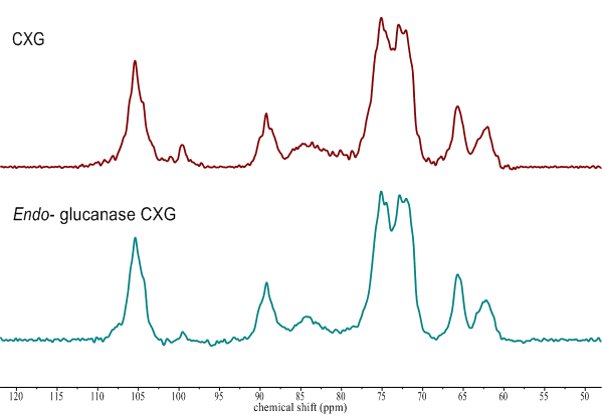

Supplement: S1 Fig — (TIF) [file pone.0122132.s001.tif]

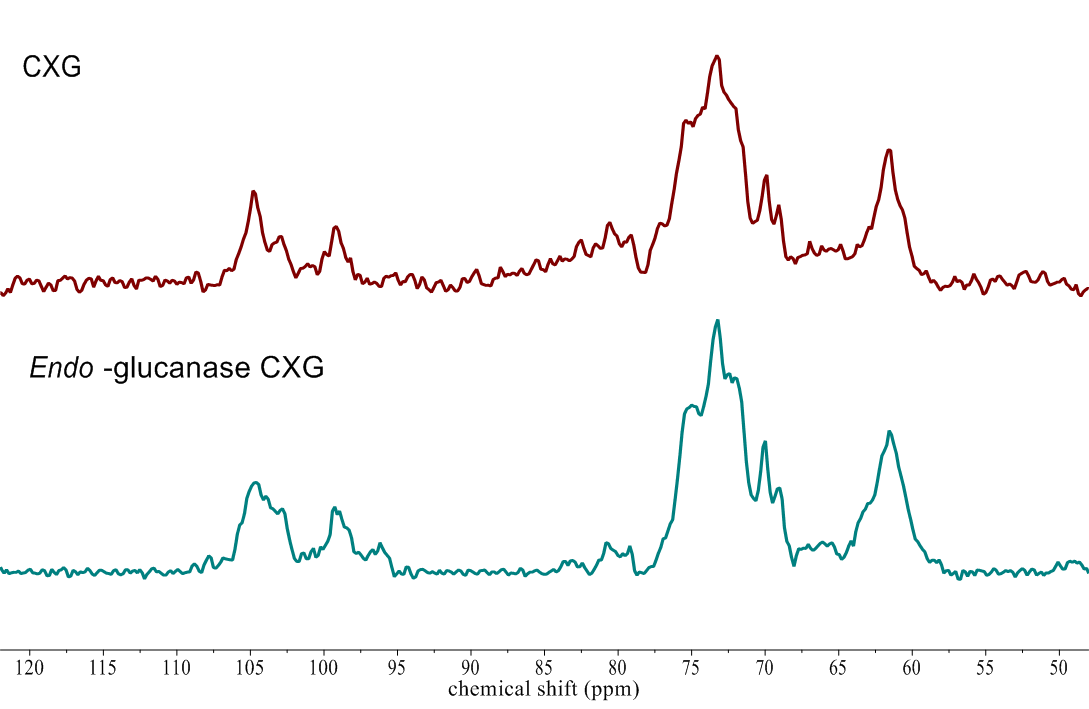

Supplement: S2 Fig — Xyloglucan still present following enzyme treatment. (TIF) [file pone.0122132.s002.tif]

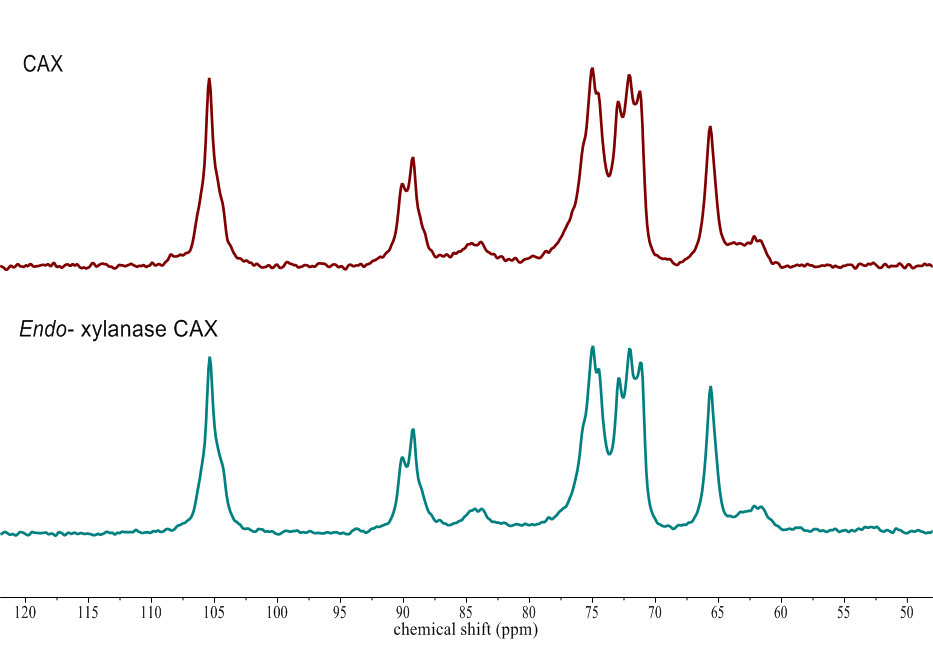

Supplement: S3 Fig — Cellulose is unchanged following enzyme treatment. (TIF) [file pone.0122132.s003.tif]

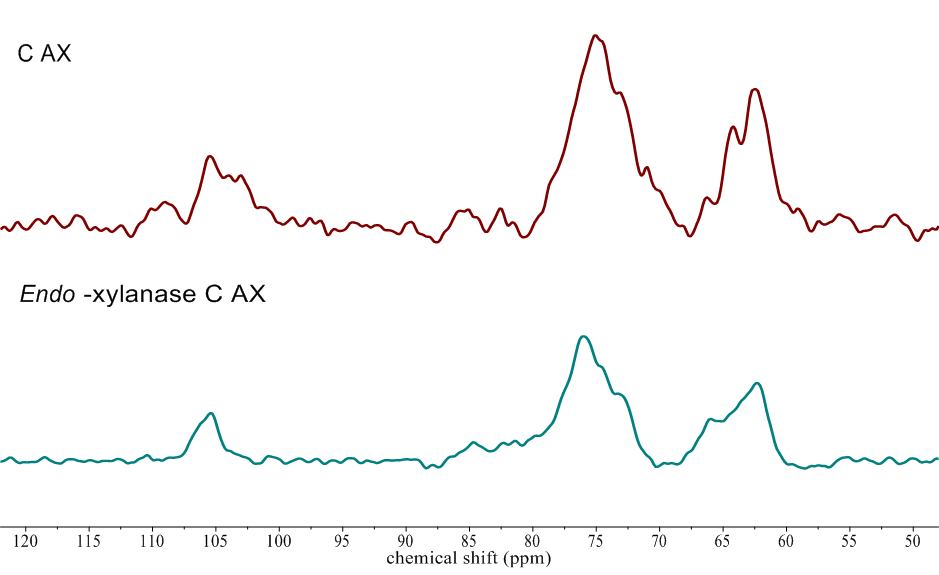

Supplement: S4 Fig — C-1 xylose peak at 102.4 ppm being completely removed and the arabinose peak at 108.5 also no longer evident. (TIF) [file pone.0122132.s004.tif]

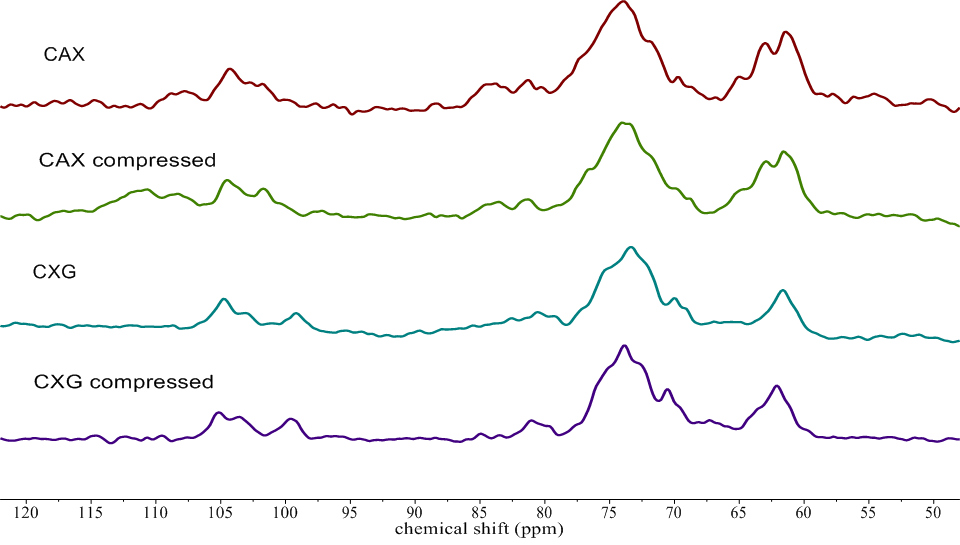

Supplement: S5 Fig — (TIF) [file pone.0122132.s005.tif]
